# Potential Role of Oxidative Stress in the Production of Volatile Organic Compounds in Obesity

**DOI:** 10.3390/antiox12010129

**Published:** 2023-01-05

**Authors:** Adebowale Samuel Oyerinde, Vaithinathan Selvaraju, Jeganathan Ramesh Babu, Thangiah Geetha

**Affiliations:** 1 Department of Nutritional Sciences, Auburn University, Auburn, AL 36849, USA; 2 Boshell Metabolic Diseases and Diabetes Program, Auburn University, Auburn, AL 36849, USA

**Keywords:** obesity, oxidative stress, volatile organic compounds, metabolites, reactive oxygen species, lipid peroxidation, inflammation

## Abstract

Obesity is associated with numerous health issues such as sleep disorders, asthma, hepatic dysfunction, cancer, renal dysfunction, diabetes, cardiovascular complications, and infertility. Previous research has shown that the distribution of excess body fat, rather than excess body weight, determines obesity-related risk factors. It is widely accepted that abdominal fat is a serious risk factor for illnesses associated with obesity and the accumulation of visceral fat promotes the release of pro-oxidants, pro-inflammatory, and reactive oxygen species (ROS). The metabolic process in the human body produces several volatile organic compounds (VOCs) via urine, saliva, breath, blood, skin secretions, milk, and feces. Several studies have shown that VOCs are released by the interaction of ROS with underlying cellular components leading to increased protein oxidation, lipid peroxidation, or DNA damage. These VOCs released via oxidative stress in obese individuals may serves as a biomarker for obesity-related metabolic alterations and disease. In this review, we focus on the relationship between oxidative stress and VOCs in obesity.

## 1. Introduction

Obesity, the major health dilemma of the twenty-first century, affects the physiological, economic, and psychological quality of life of individuals regardless of racial, financial, cultural, or societal background [1]. Excess accumulation of body fat reduces the quality of life, raises medical care costs, and increases the mortality rate. Obesity leads to numerous health challenges, including diabetes, cancer, renal dysfunction, cardiovascular complications, asthma, hepatic dysfunction, and infertility [2]. Obesity-related risk factors are governed by the distribution of excess body fat rather than excess body weight [3]. The accumulation of visceral fat promotes pro-inflammatory and pro-oxidant states and is recognized as a substantial risk element for obesity-related disorders [4]. The term “reactive oxygen species” (ROS) refers to a class of substances that are created during normal oxygen metabolism, which is having at least one unpaired electron occupying a single orbital.

Free radicals such as hydroxyl and superoxide anion radicals are examples of ROS species, along with non-radicals such as ozone, hypochlorous acid, and hydrogen peroxide. Under usual circumstances, low ROS levels have beneficial biological effects because they are involved in homeostasis and crucial molecular pathways such as redox state or modulating cellular metabolism, cell signaling, inhibiting, or activating numerous gene transcription factors, inhibiting bacterial growth, inactivating viruses, and producing inflammatory cytokines. Moreover, these rigorous environmental stress stimuli result in increased ROS levels [5]. 

Oxidative stress (OS) is a disruption in the balance between free radical generation and antioxidant defenses that can cause tissue damage. Free radical damage can build up over time and become a major contributor to a wide range of oxidative stress-related human diseases. Increased ROS concentrations are powerful inducers of cellular damage at the cellular level, specifically targeting structures such as phosphorylated lipids as components of proteins, cellular membranes, and DNA [5]. Methods have been developed to further understand the proportions of OS damage caused by various cellular OS products, such as protein carbonyls from protein oxidation, malondialdehyde as a marker for lipid peroxidation, and 8-hydroxy-20-deoxyguanosine (8OHdG) as a biomarker for DNA oxidative damage [5]. New diagnostic tools are surfacing as prospective biomarkers of these conditions. In the human body, metabolic processes release diverse volatile organic compounds (VOCs) through urine, breath, blood, saliva, skin secretions, milk, and feces [6]. VOCs are classified as organic compounds containing oxygen (acetone), sulfur (hydrogen sulfide, methyl-mercaptan, dimethyl sulfide), or nitrogen (dimethylamine, trimethylamine, ammonia), short-chain fatty acids (acetate, isobutyrate, propionate), as well as saturated and unsaturated hydrocarbons such as alkenes, alkanes, aldehydes, or ketones. It has been demonstrated in numerous studies that a metabolomics evaluation of VOCs from biological fluids might offer functional data for clinical diagnosis and treatment monitoring of several diseases including obesity, gastrointestinal problems, and cancer [7,8]. The ROS interactions with innate biological components promote protein oxidation, lipid peroxidation, or DNA damage, and several VOCs are generated. A clearer understanding of how OS and the emission of VOCs from intended substrates such as obesity, cancer, and cardiovascular disease interact would greatly aid in determining the precise origin or pathways of VOC compounds in various pathologies. In this review, we focus on the relationship between VOCs and OS in obesity.

## 2. Oxidative Stress in Obesity

**Figure 1:**
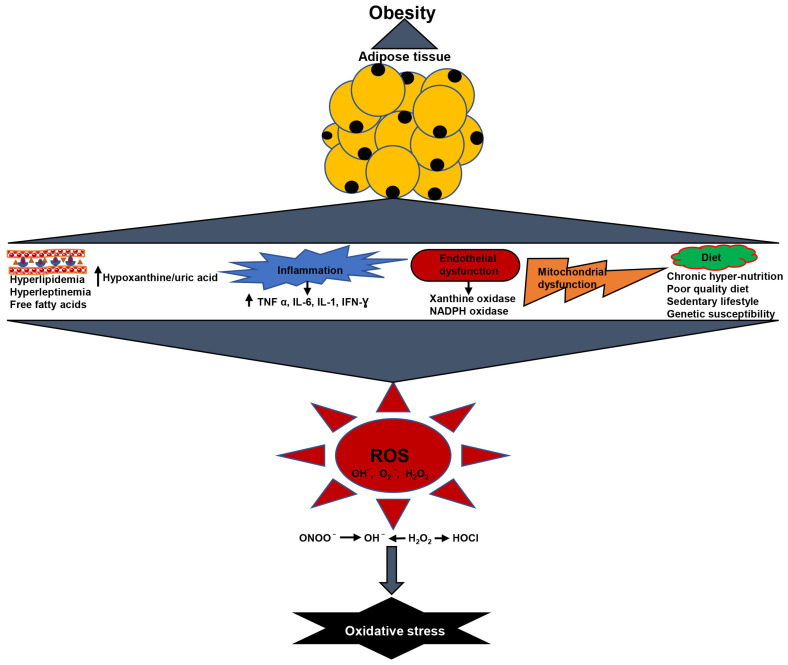
Oxidative stress generation via ROS production in obesity.

The biomarkers of systemic oxidative stress such as isoprostanes (F2-IsoP), F-2 8-isoProstaglandin F2 α (8-isoPGF2 α), malondialdehyde (MDA), and protein carbonylation detected in serum, urine, or plasma have been linked to the degree of fat storage [9]. Fat accumulation and insulin resistance increase the hepatic TCA cycle’s oxidative and anaplerotic pathways, generating more NADH and FADH_2_. This raises the proton gradient along the inner mitochondrial membrane, causing electron leakage at complex III and superoxide production [10]. The upstream metabolites are redirected into four alternative pathways due to the free radical’s inhibition of glyceraldehyde 3-phosphate dehydrogenase. The pathways include glucose being moved to the polyol pathway, fructose-6-phosphate being moved to the hexosamine pathway, triose phosphates being converted to methylglyoxal, the primary precursor of advanced glycation end products (AGE), and dihydroxyacetone phosphate being converted to diacylglycerol, which stimulates the PKC pathway [11]. Increased free radical generation or weakening antioxidant defenses, and the activation of these alternate metabolism causes OS. Hyperglycemia-induced increases in polyol pathway flux, which promotes the conversion of glucose to sorbitol by aldose reductase and nicotinamide adenine dinucleotide phosphate (NADPH), thus activating numerous stress-related genes and increasing redox stress due to NADPH consumption. Since NADPH is needed to recreate reduced glutathione (GSH), which scavenges ROS, this could increase intracellular oxidative stress [12,13]. The hexosamine pathway’s generation of glucosamine-6-phosphate limits thioredoxin activity and causes oxidative and endoplasmic reticulum stress, and AGE and PKC promote ROS or reactive nitrogen species (RNS) by activating NADPH oxidases (NOX) enzymes and NF-kB [14,15]. Superoxide radicals (O^2-^) are produced in greater amounts when NOX enzymes are activated because they catalyze the reduction of oxygen with NADPH acting as an inner electron donor [16]. Reactive oxidants, which are the same as hydroxyl and superoxide radicals, are produced by the auto-oxidation of glucose [17]. AGE reacts with certain cell surface receptors, altering post-receptor signaling and promoting the generation of more ROS [18]. NF-kB activation promotes the transcription of adhesion molecules (intercellular adhesion molecule-1, E-selectin, and endothelin-1), pro-inflammatory cytokines (IL-6 and TNF-α), microRNAs, and inducible nitric oxide synthase (iNOS) which are involved in adipogenesis, inflammation, and OS [19]. The oxidative stress generation in obesity is represented in Figure 1.

### 2.1. Obesity Is Associated with Elevated Lipid Levels and Oxidative Stress

Elevated plasma free fatty acids (FFA) elevate the production of O_2_ in the mitochondrial electron transport chain by impeding adenine nucleotide translocation in the obese individuals via PKC-dependent activation of NOX in biological cultured vascular cells [20]. Free fatty acids increase the production of reactive intermediates [21]. Conjugated fatty acids are prone to oxidation, which leads to the production of radicals and excess oxidative byproducts [22]. Obese individuals have a higher level of 4-hydroxynonenal (4-HNE) per unit of intramuscular triglycerides, implying that lipids are susceptible to oxidative modification [23]. Obesity-related lipid molecule concentrations may also occur in a larger target for oxidative modification by ROS [24]. Furukawa et al. discovered that excessive fat accumulation in white adipose tissue (WAT) leads to a rise in lipid peroxidation in the WAT itself in several obesity studies [22]. The food source of some lipids can also lead to OS. Conjugated linolenic acid from dietary intake raised the urinary level of 8-epi PGF2 α in adults with visceral fat obesity [25].

### 2.2. Chronic Low-Grade Inflammation Produced Oxidative Stress in Obesity

Obesity induces the production of common inflammatory cytokine mediators in the adipose tissue such as IL-6, TNF-α, and IL-1, which promote ROS generation by monocytes and macrophages; thus, an increase in the level of ROS could suggest increased OS [26]. Macrophages play an important role in controlling obese inflammation by shifting T-helper (Th) cell differentiation toward the Th1 subtype, a pro-inflammatory condition. The most important inducer of ROS generation by macrophages during the Th1 immune response is interferon-gamma (IFN-γ). Increased levels of ROS from macrophages, including O_2_, H_2_O_2_, and OH, also promote the activation of Th1 cells [27]. Furthermore, leptin promotes the proliferation of macrophages and monocytes, thereby promoting the generation of pro-inflammatory cytokines (IL-6 and TNF-α) [28,29]. Plasma leptin concentrations are increased in obesity [30]. Leptin prompts macrophage lipoprotein lipase activity and PKC activity in monocyte-derived macrophages [31]. Leptin also decreases the action of the cellular antioxidant paranoxase-1 (PON-1) which is linked to a higher concentration of plasma MDA and hydroperoxides, as well as plasma and urinary 8-isoPGF2 α [28].

### 2.3. Obesity Causes Increased Hypoxanthine/Uric Acid and Oxidative Stress

Increased muscle activity in obese individuals can result in an excess of free radicals due to the stimulation of metabolic pathways such as increased activity of the electron transport chain and the transformation of hypoxanthine into urate [32]. After severe exercise and hypoxia or ischemia, serum hypoxanthine levels rise. They are extracellular metabolites that indicate tissue hypoxia by monitoring intracellular energy metabolism [33]. Hypoxanthine metabolism produces free radicals [34]. Studies showed a significant increase in hypoxanthine with exercise in obese people, which may harm organs due to free radicals [32]. In obese people, strenuous exercise may cause free radical damage. Muscle cells create hypoxanthine through inosine monophosphate (IMP) during hard exercise, decreasing muscle adenine nucleotide concentration [35]. Intensity and duration of exercise increased cell adenine nucleotide elimination [36]. Hypoxanthine is slowly released into the bloodstream and taken up by the liver to be converted to uric acid [32]. 

However, increased respiration may result in leakage of some electrons from the electron transport chain due to rapid electron transfer [37]. Obese people consume more energy for a given workout load because they are less effective while exercising [38]. Obese people have higher hypoxanthine levels during exercise and the transformation of hypoxanthine to urate is related to the production of superoxide anion [32].

### 2.4. Obesity Causes Endothelial Dysfunction and Oxidative Stress

Numerous enzymatic sources of oxidant production, such as NO synthase, NOX, and xanthine oxidase are crucially located in the vascular endothelium [39]. The generation of endothelium O^2−^ is significantly aided by NOX activation [40]. O^2−^ and H_2_O_2_ are also produced by xanthine oxidase’s reaction with O_2_ [41]. Rapid formation of peroxynitrite ONOO^-^ from excessive O^2−^ production decreases the bioavailability of NO and results in protein nitrosylation [42]. The enzyme NO synthase promotes the production of excess ONOO^-^ and O^2-^ by facilitating the transfer of electron transport from NADPH to another heme group [43]. These oxidant-producing enzymes can be affected by the actions of other cytokines and hormones, most especially those in the renin–angiotensin system. Obesity is linked with higher renin–angiotensin system hormone concentrations [13]. Increased angiotensin II concentrations can stimulate OS in the vasculature through different pathways, such as NOX activation, O^2−^, and H_2_O_2_ formation [44,45]. Elevated intraluminal pressure caused by obesity-related hypertension may encourage the production of O^2−^ and ONOO^−^ [46].

### 2.5. Obesity Causes Mitochondrial Dysfunction and Oxidative Stress

Mitochondrial dysfunction is caused by the inability of mitochondria to generate and sustain sufficient ATP levels [47]. Rapid increases in mitochondrial biogenesis and activity during adipocyte differentiation imply that mitochondria play a significant role in this organelle [48]. During oxidative phosphorylation in the mitochondria, a slight excess of electrons results in a reduction in oxygen which generates potentially harmful free radicals [4]. The regulation of free radical generation in mitochondria can also change when several uncoupling proteins reintroduce protons into the mitochondrial matrix, under specific circumstances [49].

### 2.6. Diet and Oxidative Stress in Obesity

Diet may also affect how much ROS is produced by obesity and the risk factors that go along with it. A high-fat diet may change how your body metabolizes oxygen. It has been discovered that consuming a high-fat, high-carbohydrate diet causes significant oxidative stress and inflammation in obese people [50]. Reduced dietary intake of antioxidant-rich vitamins may result in insufficient antioxidant defense [51]. Adiposity, BMI, and lipid peroxidation are all negatively correlated with dietary consumption of antioxidant phytochemicals [51]. It was shown that obese people had lower serum concentration of dietary antioxidants and trace minerals (zinc, selenium, etc.) that serve as cofactors for antioxidant enzymes, than non-obese people. 

## 3. Volatile Organic Compounds in Obesity

Some VOCs found in obese individuals include alcohols, short chain fatty acid, saturated hydrocarbons, and unsaturated hydrocarbons (Table 1). Potential routes for endogenous formation of straight chain and branched saturated hydrocarbons in mammals are reported as lipid peroxidation, protein oxidation, and intestinal bacterial metabolism [52,53]. Enhanced liver oxidative activity or increased bacterial metabolism in the ruminant gut following meal consumption may both contribute to increased lipid peroxidation. 

### 3.1. Volatile Organic Compounds in Biological Samples 

One of the most recent approaches in metabolomics for disease research appears to be the analysis of VOCs emitted from biological samples. Breath, human urine, saliva, feces, blood, human sperm, and cell cultures have all been tested for the presence of volatile markers. VOCs are a broad category of tiny, stable, lipophilic compounds with molecular weights between 50 and 200 Dalton that are volatile at room temperature [62]. All biological matrices have a volatile composition that is made up of endogenous and exogenous compounds. Endogenous molecules are those synthesized by cell metabolism (in vivo or in vitro) and may be used to identify pathological, physiological, and disease states. Exogenous VOCs are derived from environmental exposures [63,64]. Endogenous organic compounds from cell culture or biofluids can aid in understanding physio–pathological mechanisms of the root cause of diseases such as bacterial infection [65,66] and inflammation [67,68]. Exogenous, on the other hand, are crucial in exposome research for monitoring the health-related effects of other external elements, learning about pharmacokinetics, and evaluating the impact of the environment on human health. Metabolic alterations happen often throughout both healthy and unhealthy metabolic processes in the body. Such aberrant activities might affect the body’s metabolomics in metabolic diseases such as obesity by changing the level of VOCs or by creating new VOCs.

OS and the stimulation of cytochrome P-450 enzymes (CYP450, a group of oxidase enzymes) are linked to the main pathway in the creation of volatile organic molecules in a disease state [69]. ROS buildup in tissues causes various attacks on biological molecules such as polyunsaturated fatty acids (PUFA) and proteins. Free radicals and ROS are ejected from the mitochondria when a cell experiences oxidative stress, producing volatile alkanes that are released in the breath [70]. Additionally, in human tissue, ROS molecules can activate the CYP450 that catalyze the oxidation of organic substances [71,72]. In human adipose tissue, breast cancer tissue, and other tissues, it has been discovered that this enzyme family is overexpressed [73]. Keep in mind that most inflammatory disorders are linked to the formation of ROS, and obesity is an example of low-grade chronic inflammation. 

### 3.2. The Biochemical Pathway of VOCs 

Different VOCs transmit distinct body compartments-specific information in diverse ways. The ability of the human body to store numerous volatile chemicals is incredibly rare. Furthermore, the period required to deplete a compound’s stores vary. Hydrocarbons, ketones, primary and secondary alcohols, aldehydes, and branched aldehydes, esters, nitriles, and aromatic compounds (benzene derivative and others) are among the chemical families of human VOCs that undergo different metabolic pathways for their production (Table 2).

Hydrocarbons—Oxidative stress is the main process that affects the body’s generation of hydrocarbons. PUFA, which are mostly found in adipose tissue, cellular and subcellular membranes are primarily used to create alkanes by peroxidation (lipid peroxidation). Tissue injury occurs in vivo because of lipid peroxidation [70]. Saturated hydrocarbons, including pentane and ethane are produced because of lipid peroxidation. Ethane and pentane in the breath are examples of in vivo non-invasive lipid peroxidation markers that are frequently utilized [74]. Even though the existence of other saturated hydrocarbons, such as C3-C11, can be linked to the lipid peroxidation pathway, it seems that branched hydrocarbons do not really benefit from this mechanism. Hydrocarbons that are poorly soluble in blood and are not digested by the body are promptly expelled through the breath, urine, and other bodily fluids [75,76].

Alcohols—Alcohols are predominantly absorbed into the blood via diffusion from all sections of the gastrointestinal tract. The metabolism of hydrocarbons can also produce alcohols. Due to their great affinity for water, short-chain alcohols are easily entered into the bloodstream. Confounding elements in the body affect alcohol metabolism, namely variations in water and fat content between individuals and genders, can have an impact on how the body processes alcohol [70]. Alcohol metabolism in the body may be regulated by enzymes such as CYP450 (CYP2E1, of which the liver is the primary location) and alcohol dehydrogenase (ADH). Humans can oxidize a variety of alcohols through the action of ADH and any residual VOCs are eliminated through excretion of alcohol through sweat, feces, urine, breath, saliva, and breast milk [70].

Aldehydes—The body creates aldehydes as an essential component of physiological function. Aldehydes are crucial for a variety of physiological processes, and some of them are cytotoxic intermediates that have a variety of functions in signal transmission, cellular proliferation, and gene regulation [77,78]. Aldehydes may be produced in the body through various pathways such as alcohols metabolism, the reduction of hydroperoxide by CYP450 as a byproduct of lipid peroxidation [79], and the production of aldehydes as secondary oxidation products; these pathways are a major source of aldehydes. CYP450 is involved in the monofunctional C3-C10 aldehydes such as n-nonanal, n-heptanal, n-hexanal, and n-decanal synthesis from the lipid oxidation of omega-3 and -6 PUFAs, such as arachidonic acid or linoleic [80,81]. The phase I metabolic process of a wide variety of substances depends on CYP450 enzymes. They convert these molecules to a hydrophilic state, making excretion easier [82,83]. Smoking contributes to the presence of aldehydes in the body. For example, formaldehyde, acetaldehyde, and acrolein are saturated and unsaturated aldehydes that are present in tobacco smoke [84], and the detoxification process is carried out by CYP450 because of the byproduct of tobacco metabolism [85,86]. Additionally, aldehydes can also come from food sources [87,88].

Ketones—Ketone bodies are produced when the rate of fatty acid oxidation accelerates because of modification in metabolic circumstances [89]. β-hydroxybutyrate and Acetoacetate, which are produced in huge quantities by the liver, can also undergo non-enzymatically decarboxylated to yield acetone. Acetone is the least abundant of the ketone bodies and can be expelled by the skin, urine, and breath because of its high vapor pressure. Ketone bodies can also be produced through the metabolism of proteins. In the state of Cachexia, which is caused by an increase in protein metabolism, which raises ketone body levels, acetone levels can be altered via eating, exercise, and fasting [90,91]. Furthermore, ketone synthesis from other external sources, such as food or environmental pollutants, may eventually be taken up by the body [92,93].

Esters—Esters are a class of substances that are abundantly present in fruit, essential oils, natural wax, fatty acids and lipids. In humans, at temperatures lower than 40 °C, esterase hydrolyzes esters into acid and alcohol [94]. Lipase is one common illustration of esterase enzymes, which catalyzes the breakdown of lipids as a byproduct of the body’s regular metabolic process.

Nitriles and aromatic compounds—Typically, nitriles and aromatic VOCs are regarded as exogenous source contaminants, including exposure to pollution, radiation, smoking, and alcohol [70]. High reactivity of these compounds causes peroxidative damage to PUFA, DNA, and proteins [95]. These chemicals are kept in the body’s adipose tissues. A more excretable and soluble version of the molecule is produced because of the two-phase elimination of dangerous compounds and xenobiotics by cellular, mechanical, and enzymatic defense mechanisms [70,96]. Acetonitrile is one such substance that is present in smokers. The mechanism proposed for acetonitrile is the biotransformation of cyanohydrin to hydrogen cyanide and formaldehyde by CYP450 monooxygenase which can be excreted via exhaled breath and/or urine [70,97]. 

### 3.3. Analytical Techniques for VOCs Detection

Gas chromatography linked with mass spectrometry (GC-MS) has been widely acknowledged as the ultimate standard in VOCs detection with the attachment of various pre-concentration techniques such as SPME (solid phase microextraction), NTDs (needle trap devices), or TD (thermo-desorption). Each preconcentration method has a unique set of traits. TD offers great sensitivity and consistently produces quantitative data, whereas NTD is adaptable but has additional calibration procedures. SPME is straightforward to use but has a low sensitivity and is only semi-quantitative. Direct mass spectrometric methods include proton-transfer-reaction time-of-flight mass spectrometry (PTR-TOF-MS), selected ion flow tube mass spectrometry (SIFT-MS), secondary electrospray ionization mass spectrometry (SESI-MS, SESI-Q-TOF), and proton-transfer-reaction mass spectrometry (PTR-MS). These techniques can analyze samples considerably more quickly than GC-MS since they do not split the volatile chemicals before analysis.

## 4. Interconnection of Volatile Organic Compounds and Oxidative Stress in Obesity

The production of aldehydes and hydrocarbons is typically attributed to increased OS [98], which denotes an imbalance between the formation of ROS and the antioxidant capacity of the organism [99]. The OS may lead to an excess of ROS, such as hydroxyl radical (OH^−^), hydrogen peroxide (H_2_O_2_), and superoxide anion (O^2−^). Adipogenesis is always accompanied by an elevated OS concentration and the activation of CYP450 [100], which leads to the lipid peroxidation of PUFA in adipose tissue [101,102]. Then, the volatile aldehyde, hydrocarbon, and hydrocarbon-derivatives can be produced and excreted via exhaled breath, urine, skin, blood, etc. [101,102].

Many potential VOCs have been proposed successively as oxidative stress biomarkers, such as 2-propanol [103], formaldehyde [104], and the markers of octanal, hexanal, nonanal, and heptanal [105]. Elevated lipid peroxidation produced hydrocarbon. Alkanes, aldehydes, and ketones synthesized by lipid peroxidation are suggested to be the major pathway of VOC changes in exhaled breath diagnosis [98]. Linear chain alkanes such as N-dodecane and hexane, are shown to be present in higher amounts in the OS. According to research, alkanes in the breath are frequently suggested as OS byproducts [53,98,106]. Saturated hydrocarbons, such as C3–C11, may be produced due to the lipid peroxidation pathway. However, in the presence of branched hydrocarbons, this mechanism is probably ineffective. Hydrocarbons that cannot be digested and absorbed in the body are eliminated through the urine, blood, and breath. The solubility of volatile hydrocarbons in diverse cellular mediums affects the concentrations of these substances in biological matrices. Poor solubility in the blood hydrocarbons quickly escaped into the breath. According to Kneepkens et al. [107] ROS might change n-carbon atoms PUFAs into lipid alkoxy groups, which would then decompose to produce n-1 carbon atoms alkanes. Ethane and pentane, respectively, were produced by the conversion of the ω3 and ω6 fatty acids. Other alkanes such as propane, hexane, butane, heptane, and octane could also be produced via the method. It has been reported that OS can raise the levels of branched chain alkanes such as 3-ethylhexane, 2,6,10-trimethyltetradecane, 2,3,5,8-tetramethyldecane, 5-methyltridecane, and 2,4-dimethyleicosane [108]. Hydrocarbons such as 1-octene and 1-decene are thought to be oxidative stress biomarkers in obesity [80,109]. Acrolein is produced endogenously from lipid peroxidation during OS, as well as catabolism of threonine and methionine can lead to the observed levels [110]. According to a review by Moghe et al. [111], acrolein influences metabolic pathologies through several mechanisms and target tissues, such as oxidative stress induction, endoplasmic reticulum (ER) stress, protein adduction, inflammation, mitochondrial dysfunction, immune changes, deregulated signal transduction, and structural and membrane effects. As a result, it might be a useful breath resource for tracking obesity and carbonyl stress [111]. The link between branched chain alkanes and linear chain alkanes is not yet fully understood theoretically. Furthermore, Phillips et al. [53] hypothesized that while OS rose with age, branched chain alkanes and linear chain alkanes in exhaled breath also increased. Branched chain alkanes can thus also be an outcome of OS. The branched chain alkanes, however, may undergo intricate chemical processes in cells, tissues, or even complete organisms. Due to the absence of branched unsaturated fatty acids in cells, the opposing theory contends that branched chain alkanes may not be the end-product of lipid peroxidation [112,113].

ROS can be produced due to reactions initiated by CYP450s, which are overabundant in obesity, diabetes, and cancer cells. The CYP450 family 1B1 gene has been shown to be overexpressed in many types of tumor cells and has been linked to angiogenesis [114]. Another member of the CYP450 family, CYP2E1, is one of the main potent enzymes in terms of ROS production. They cause ROS production, which results in unfolded protein response, autophagy, enhanced angiogenic responses, DNA damage, and ER stress. Exogenous (e.g., ethanol, pyrazole, isoniazid) or endogenous (e.g., obesity, diabetes, fasting, hypophysectomy) factors can both increase CYP2E1 activity [115]. It has been demonstrated that animals with diet-induced obesity express more hepatic CYP2E1 protein and better metabolize typical CYP2E1 substrates [116]. A higher number of inflammatory cytokines in the adipose microenvironment is caused by overexpression of the CYP2E1 gene in morbidly obese people compared to non-obese people, and this raises hepatocellular CYP2E1 expression in those with steatohepatitis [117]. Therefore, it has been proposed that CYP2E1 upregulation and CYP2E1-mediated oxidative damage play a major part in the mediation of steatohepatitis linked with morbid obesity [118]. CYP2E1-generated ROS can support the growth of tumors in several ways [115]. Alkanes are largely generated by the lipid peroxidation of the PUFAs found in adipose tissue, which leads to phospholipid degradation and, ultimately, cellular deterioration [53,107]. Aldehyde can also produce alkanes in the context of hepatic ethanol metabolism. Several alkanes such as ethane and pentane have been found to be elevated in obese people but only pentane is much more elevated in obesity. Oxidative stress can be estimated using breath measurements of biomarkers such as ethane, ethylene, and pentane [119,120]. Although the amount of these hydrocarbons in total peroxidized PUFA is tiny and probably changeable, their detection in exhaled breath enables in vivo evaluation of oxidative stress [52]. Aghdassi and Allard measured oxidative stress in several inflammatory conditions, including obesity, by measuring breath alkane output and other markers of lipid peroxidation [119]. Obese people had significantly higher lipid peroxidation and significantly lower antioxidant vitamin status when compared to non-obese people. According to Allard et al. in an animal study of vitamin E deficiency, increased peroxidation of tissue lipids causes an increase in breath pentane [121]. However, Gelmont et al. suggest that dietary linoleate was also necessary for pentane synthesis [122]. Breath pentane was mostly produced by gut bacteria in addition to endogenous membrane lipid peroxidation [122]. However, alterations in the lipid composition of membranes and raised oxidative stress in tissue cells may be responsible for increased aldehyde generation in obese people. Moreover, increased amounts of unsaturated fatty acids in adipose cell membranes may boost the lipid peroxidation process’ ability to produce certain aldehydes. In contrast, ROS in the cells may increase the activity of the CYP450 and encourage the conversion of alkanes to alcohols [71,72]. However, excessive aldehydes from lipid oxidation under OS could turn into carboxylic acids by additional oxidation. As a result, the production of alcohols and carboxylic acids under OS would encourage the formation of esters.

Although the metabolism of lipids and amino acids, which are thought to be the main likely sources of VOCs, significantly shift when cells are under OS, in order to link volatile metabolites to intracellular metabolic pathways, more research is necessary. For instance, a metabolic pathway that demonstrates the conversion of phenylalanine to phenylethylamine. Of course, there might be other unintended causes as well, including how powerful OS regulates the metabolism of the entire cell and activates antioxidant enzymes. Previous research has proven that there is derangement in glucose and fatty acid metabolism in obese adipose tissue which exhibit hyperlipolytic activity, resulting in excess free fatty acids and glycerol [123,124]. These excess lipid, fatty acids, and glycerol are easily attacked by free oxygen radicals or ROS to produce volatile hydrocarbon through the process of lipid peroxidation. The hypertrophied adipocytes tissue also produced free volatile fatty acids or its esters derivatives [123,124], although more research is needed to establish this statement. 

Increased mitochondrial enzyme activity is associated with adipogenic development, which denotes an increase in oxidative phosphorylation and oxidation capability and, consequently, a shift towards lipid metabolism [125]. The primary source of ATP in humans is the TCA cycle in the mitochondrion. In contrast to skeletal muscle and liver, obese adipose tissue grows due to energy oversupply [126,127,128,129]. Due to the high levels of oxidative phosphorylation in adipose mitochondria, either an excess of released electrons results in a decrease in oxygen, which creates potentially harmful free radicals [4], or several uncoupling proteins can reintroduce protons into the mitochondrial matrix, which alters how free radical production in mitochondria is regulated [49]. These ROS produced during oxidative stress also attacked DNA and protein apart from lipid which led to protein and DNA damage. These ROS attacked amino acids molecules of the protein and nucleic acids molecules of the DNA which led to production of small fragments of molecules that are volatile in nature. According to a recent study, obese mice produce more uric acid than normal because their adipose tissue secretes more glutamate [130]. The liver or skeletal muscle did not exhibit these metabolic alterations. Excessive uric acid synthesis in obese adipose tissue may be linked to accelerated purine metabolism due to ROS attack on the DNA and protein which may be accompanied by production of some volatile compounds such as 2,4,6-trimethyl-pyridine, triethylamine, and trimethylamine etc. Previous studies reported that amino acids, particularly isoleucine, and leucine are vulnerable to the generation of volatile hydrocarbons when attacked by ROS, and antioxidant enzymes can prevent the formation of VOCs [113]. As a result, OS exposure causes aberrant amino acid metabolism, which is a significant source of volatile indicators. VOCs can be directly produced from amino acids and fatty acids [98,113].

Furthermore, if OS affected other significant intracellular metabolisms, including the TCA cycle and glycogen metabolism, energy and intracellular metabolic intermediates might be impacted. The volatile metabolites may change in relation to the overall adipose tissue metabolism, but more research is required to prove the VOCs and their metabolic pathways. Studies have also shown an increase in dihydroxyacetone phosphate and citrate biosynthesis in obese adipose tissue [108], which could be a source of some volatile compound such as acetone. Isoprene is a cholesterol biosynthesis byproduct that may be overexpressed in obese people [131]. In fact, the metabolism of cholesterol in obese individuals with fatty liver disease is characterized by increased isoprene synthesis and decreased absorption [132]. Additionally, research points to the possibility of isoprene production by the gut flora [133]. It is widely known that obese individuals with liver dysfunction have higher levels of ammonia and sulfur-containing chemicals [134]. Fatty liver disease has 40–50% prevalence in obese children and adolescents which may help to explain some of the alterations in hydrogen sulfide and ammonia levels [135,136]. Aromatic VOCs found in exhaled breath were once assumed to result from exogenous substances such as cigarettes [137]. 

**Figure 2:**
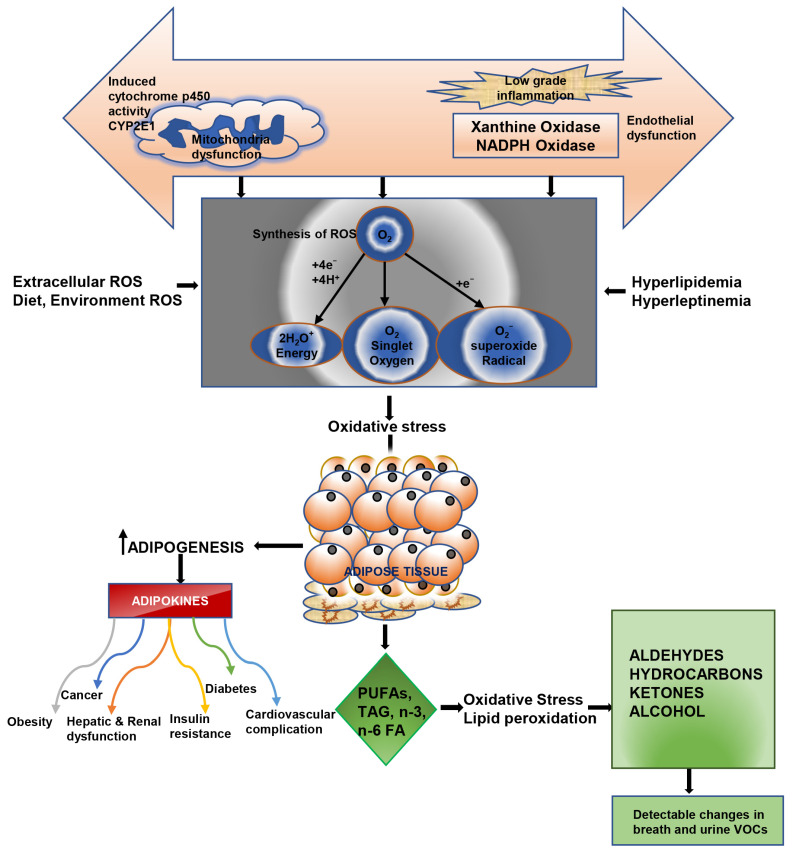
Model showing interconnection between oxidative stress and volatile organic compounds production in obesity.

It has been hypothesized that hydrocarbons (alkane, alkene, and alkyne) are produced when lipids are peroxidized by the action of OS caused by ROS. CYP450 will further oxidize these hydrocarbons to alcohols [119], and then into aldehydes and carboxylic acids, respectively, by alcohol dehydrogenase and aldehyde dehydrogenase [138]. The interconnection between oxidative stress and volatile organic compounds production in obesity is represented in Figure 2. 

### Potential Role of Oxidative Stress-Generated VOC in Obesity-Related Problem 

VOC alterations in patients with chronic renal disease and diabetes have been detected in recent investigations [139]. One of the leading causes of both cardiovascular disease and type 2 diabetes mellitus is obesity. Further, it has been connected to renal dysfunction [140]. Obesity has been shown to create persistent low-grade inflammation and to generate oxidative stress, both of which play a role in the systemic metabolic dysfunction that is connected to obesity-related illnesses [141]. As a result, a metabolic flip or dysregulated metabolism in adipose tissue might be detected early and simply by VOC profiling, which could speed up and improve the sensitivity of clinical diagnosis. There may be a correlation between the altered release of VOCs from adipocytes and the breath VOCs of diabetic patients [142], which is related to the metabolic connection between diabetes, obesity, and adipose tissue. It has been postulated that in the presence of an abundance of energy (as is the case in the obese state), lipogenesis is triggered by a metabolic switch that allows adipocytes to survive and sustain stress episodes such as hypoxia [143]. Long term, this may explain the escalating nature of obesity, metabolic shifts, and the emergence of metabolic diseases. Indeed, extensive microarray analysis has discovered strong links between the gene expression pattern of human adipocytes and matching indicators for metabolic disorders such as diabetes and insulin resistance [143]. Unfortunately, human adipocytes’ VOCs profiles have not been studied.

VOCs have been linked to obesity in several studies. Alkhouri et al. [57] discovered that isoprene-1decene, 1-octene, ammonia, and hydrogen sulfide were significantly higher in fat people than lean people, which explains that overweight children’s exhaled VOCs can be used to screen for obesity-related comorbidities and study the epidemic’s causes and mechanisms. Obesity-related liver problems are examined through breath analysis. Alkhouri et al. [58] examined exhaled breath analysis for pediatric diagnosis. SIFT-MS breath analysis was used to distinguish obese children with and without nonalcoholic fatty liver disease (NAFLD). They found that isoprene, acetone, trimethylamine, acetaldehyde, and pentane may identify NAFLD children from others.

Solga et al. [144] compared breath indicators to blood serum tests for diagnosing NAFLD patients, some of whom had nonalcoholic steatohepatitis (NASH). Patients with severe steatosis (grade 2 or 3), steatohepatitis, and NASH exhibited greater breath acetone levels than those with lesser forms. Breath ethanol also increased with hepatic steatosis severity (grade 2 and 3).

Raman et al. [145] investigated VOCs trends in feces between obese and NAFLD patients and healthy controls using GC-MS. A core group of ester VOCs was higher in obese NAFLD patients than healthy controls (normal liver and lean). Ester compounds dominated VOCs (i.e., aliphatic esters of ethanoic, butanoic, propanoic, and pentanoic acids) and they are short-chain aliphatic alcohols and carboxylic acid derivatives compounds.

## 5. Conclusions

The current review discusses how volatile organic compounds can be produced during oxidative stress in obese individuals. Overall, it can be suggested that the exact metabolic mechanisms for VOCs are gradually unfolding. This review has highlighted that VOCs are generated by the peroxidation of lipids due to the action of ROS as a result of OS, and they will be further oxidized into alcohols by CYP450, which can be further oxidized into aldehydes and carboxylic acids by alcohol dehydrogenase and aldehyde dehydrogenase. Then, as is widely believed, adipose tissue might play a more direct role in metabolic regulation and detoxifying processes. Adipose tissue regulates lipid mobilization because it serves as a fuel storage. Changes in the human volatilome are therefore reflective of adipose tissue malfunction and may be identified non-invasively using breath or urine tests. VOCs produced during lipid peroxidation may serve as useful indicators of this metabolic status. However, the findings show a mixed result, because VOCs can also be produced via colonic and gut bacterial metabolism. Hence, more future research needs to be conducted using both human and cellular models. This will help to elucidate the precise origin of VOC compounds in obese individuals which can be used as a diagnosis tool in the disease prognosis.

## Figures and Tables

**Table 1:** Volatile organic compounds evident in obese individuals.

No.	Volatile Metabolite	Study	Method	Fluid
1	5-methyl-3-hexanone, 1-heptanol, 4-methyl-2-heptanone, 2-hexanol, dimethyl sulfone, formamide N, N-dibutyl, 1-hexanol, 2-pentanone, 2,4,6-trimethyl-pyridine, 3-hexanone, 3-octanone, 2,4,4-trimethyl-1-pentanol	Cozzolino et al. [54]	SPME and GC-MS	Human urine
2	Acetic acid, methanol, carbon dioxide, (methylthio)methanethiol, acrolein, methylacetate, ammonia, fragments of aldehyde (butanal, hexanal, octanal or nonanal), acetone, propanol	Kistler et al. [55]	Proton-transfer reaction time-of-flight mass spectrometry (PTF-MS)	Mouse breath
3	Methanol, methylacetate, propionate, dimethyl disulfone	Kistler et al. [56]	PTF-MS	Mouse breath
4	Acetaldehyde, acetone, isoprene, 1-decene, 1-octene, ammonia, hydrogen sulfide	Alkhouri et al. [57]	SIFT-MS	Human breath
5	2-propanol, acetaldehyde, acetone, acrylonitrite, benzene, carbon disulfide, dimethylsulfide, ethanol, isoprene, pentane, 1-decene, 1-heptane, 1-nonene,1-octene, 3-methylhexane, 2-nonene, ammonia, ethane, hydrogen sulfide, triethylamine, trimethylamine	Alkhouri et al. [58]	SIFT-MS	Human breath
6	Acetaldehyde, acetone, 2-methyl-butanal, 3-methyl butanal, 5-octadecene, 3-methyl butanol, 1-pentanol, methylpyrazine, 2,6 dimethyl pyrazine, dimethylsulfide, nonanal, methional, 3-octadecene, phenol	Uchikawa et al. [59]	Headspace sampler GC-MS	Mouse feces
7	Acetaldehyde, pentane, 1,3-bis-(1,1- dimethylethyl) benzene, ethylbenzene, benzaldehyde, heptanal and octanal	Klemenz et al. [60]	Needle trap micro extraction and GC-MS	Adipogenically differentiated mesenchymal stromal/stem cells from human adipose tissue
8	Tetrachloroethane, 2,3,5 trimethyl-hexane, beta-pinene, 1,3,5 trimethyl benzene, 9-methyl acridine, tetradecane, 6,10 dimethyl-5,9 undecadien-2-one, beta-ionone	Dragonieri et al. [61]	Electronic nose TD-GC-MS	Human breath

**Table 2:** Biological VOCs and potential metabolic pathway.

No.	Class of VOC	Metabolic Pathway	Examples of the VOC
1	Hydrocarbon	Lipid peroxidation	Ethane, pentane, decane, hexane, dodecane, branched chain: 3-ethyl hexane, tetradecane, tridecane. 2,4 dimethyl eicosane
2	Alcohol	Production of hydrocarbon metabolism, alcohol metabolism by alcohol dehydrogenase and cytochrome p450 (CYP2E1)	Cyclohexanol, propanol, 1-decanol, 3-octanol
3	Aldehydes	Alcohol metabolism, secondary product of lipid peroxidation, detoxification process by cytochrome p450, smoking and diet	Nonanal, decanal, butanal, pentanal
4	Ketones	Fatty acid oxidation and protein metabolism	Heptanone, acetone, 2-pentanone, 4-octanone
5	Esters	Lipid hydrolysis	Isopropylmyristate
6	Nitriles and aromatic compounds	Exogenous origin that are highly reactive resulting in peroxidative damage to PUFA, DNA, and protein.	Acetonitrile

## References

[1] Zhang Z.Y., Wang M.W. (2012). Obesity, a health burden of a global nature. Acta Pharmacol. Sin..

[2] Pi-Sunyer F.X. (1991). Health implications of obesity. Am. J. Clin. Nutr..

[3] Despres J.P., Moorjani S., Lupien P.J., Tremblay A., Nadeau A., Bouchard C. (1990). Regional distribution of body fat, plasma lipoproteins, and cardiovascular disease. Arteriosclerosis.

[4] Fernández-Sánchez A., Madrigal-Santillán E., Bautista M., Esquivel-Soto J., Morales-González Á., Esquivel-Chirino C., Durante-Montiel I., Sánchez-Rivera G., Valadez-Vega C., Morales-González J.A. (2011). Inflammation, Oxidative Stress, and Obesity. Int. J. Mol. Sci..

[5] Calenic B., Miricescu D., Greabu M., Kuznetsov A.V., Troppmair J., Ruzsanyi V., Amann A. (2015). Oxidative stress and volatile organic compounds: Interplay in pulmonary, cardio-vascular, digestive tract systems and cancer. Open Chem..

[6] de Lacy Costello B., Amann A., Al-Kateb H., Flynn C., Filipiak W., Khalid T., Osborne D., Ratcliffe N.M. (2014). A review of the volatiles from the healthy human body. J. Breath Res..

[7] Kwak J., Preti G. (2013). Challenges in the Investigation of Volatile Disease Biomarkers in Urine. Volatile Biomark..

[8] Buljubasic F., Buchbauer G. (2015). scent of human diseases: A review on specific volatile organic compounds as diagnostic biomarkers. Flavour Fragr. J..

[9] Manna P., Jain S.K. (2015). Obesity, Oxidative Stress, Adipose Tissue Dysfunction, and the Associated Health Risks: Causes and Therapeutic Strategies. Metab. Syndr. Relat. Disord..

[10] Satapati S., Sunny N.E., Kucejova B., Fu X., He T.T., Mendez-Lucas A., Shelton J.M., Perales J.C., Browning J.D., Burgess S.C. (2012). Elevated TCA cycle function in the pathology of diet-induced hepatic insulin resistance and fatty liver. J. Lipid Res..

[11] Pennathur S., Heinecke J.W. (2004). Mechanisms of oxidative stress in diabetes: Implications for the pathogenesis of vascular disease and antioxidant therapy. Front. Biosci..

[12] Giacco F., Brownlee M. (2010). Oxidative stress and diabetic complications. Circ. Res..

[13] Egan B.M., Greene E.L., Goodfriend T.L. (2001). Insulin resistance and cardiovascular disease. Am. J. Hypertens..

[14] Diaz-Meco M.T., Moscat J. (2012). The atypical PKCs in inflammation: NF-kappaB and beyond. Immunol. Rev..

[15] Piperi C., Adamopoulos C., Dalagiorgou G., Diamanti-Kandarakis E., Papavassiliou A.G. (2012). Crosstalk between advanced glycation and endoplasmic reticulum stress: Emerging therapeutic targeting for metabolic diseases. J. Clin. Endocrinol. Metab..

[16] Zhang H., Schmeisser A., Garlichs C.D., Plotze K., Damme U., Mugge A., Daniel W.G. (1999). Angiotensin II-induced superoxide anion generation in human vascular endothelial cells: Role of membrane-bound NADH-/NADPH-oxidases. Cardiovasc. Res..

[17] Aronson D., Rayfield E.J. (2002). How hyperglycemia promotes atherosclerosis: Molecular mechanisms. Cardiovasc. Diabetol..

[18] Baynes J.W. (1991). Role of oxidative stress in development of complications in diabetes. Diabetes.

[19] Bondia-Pons I., Ryan L., Martinez J.A. (2012). Oxidative stress and inflammation interactions in human obesity. J. Physiol. Biochem..

[20] Bakker S.J., RG I.J., Teerlink T., Westerhoff H.V., Gans R.O., Heine R.J. (2000). Cytosolic triglycerides and oxidative stress in central obesity: The missing link between excessive atherosclerosis, endothelial dysfunction, and beta-cell failure?. Atherosclerosis.

[21] Inoguchi T., Li P., Umeda F., Yu H.Y., Kakimoto M., Imamura M., Aoki T., Etoh T., Hashimoto T., Naruse M. (2000). High glucose level and free fatty acid stimulate reactive oxygen species production through protein kinase C--dependent activation of NAD(P)H oxidase in cultured vascular cells. Diabetes.

[22] Furukawa S., Fujita T., Shimabukuro M., Iwaki M., Yamada Y., Nakajima Y., Nakayama O., Makishima M., Matsuda M., Shimomura I. (2004). Increased oxidative stress in obesity and its impact on metabolic syndrome. J. Clin. Investig..

[23] Russell A.P., Gastaldi G., Bobbioni-Harsch E., Arboit P., Gobelet C., Deriaz O., Golay A., Witztum J.L., Giacobino J.P. (2003). Lipid peroxidation in skeletal muscle of obese as compared to endurance-trained humans: A case of good vs. bad lipids?. FEBS Lett..

[24] Vincent H.K., Powers S.K., Dirks A.J., Scarpace P.J. (2001). Mechanism for obesity-induced increase in myocardial lipid peroxidation. Int. J. Obes. Relat. Metab. Disord..

[25] Basu S., Riserus U., Turpeinen A., Vessby B. (2000). Conjugated linoleic acid induces lipid peroxidation in men with abdominal obesity. Clin. Sci..

[26] Fonseca-Alaniz M.H., Takada J., Alonso-Vale M.I., Lima F.B. (2007). Adipose tissue as an endocrine organ: From theory to practice. J. Pediatr. (Rio J.).

[27] Huang C.J., McAllister M.J., Slusher A.L., Webb H.E., Mock J.T., Acevedo E.O. (2015). Obesity-Related Oxidative Stress: The Impact of Physical Activity and Diet Manipulation. Sports Med. Open.

[28] Beltowski J., Wojcicka G., Jamroz A. (2003). Leptin decreases plasma paraoxonase 1 (PON1) activity and induces oxidative stress: The possible novel mechanism for proatherogenic effect of chronic hyperleptinemia. Atherosclerosis.

[29] Parola M., Marra F. (2011). Adipokines and redox signaling: Impact on fatty liver disease. Antioxid. Redox Signal..

[30] German A.J., Ryan V.H., German A.C., Wood I.S., Trayhurn P. (2010). Obesity, its associated disorders and the role of inflammatory adipokines in companion animals. Vet. J..

[31] Maingrette F., Renier G. (2003). Leptin increases lipoprotein lipase secretion by macrophages: Involvement of oxidative stress and protein kinase C. Diabetes.

[32] Saiki S., Sato T., Kohzuki M., Kamimoto M., Yosida T. (2001). Changes in serum hypoxanthine levels by exercise in obese subjects. Metabolism.

[33] Sorlie D., Myhre K., Saugstad O.D., Giercksky K.E. (1982). Release of hypoxanthine and phosphate from exercising human legs with and without arterial insufficiency. Acta Med. Scand..

[34] Sahlin K., Ekberg K., Cizinsky S. (1991). Changes in plasma hypoxanthine and free radical markers during exercise in man. Acta Physiol. Scand..

[35] Harkness R.A., Simmonds R.J., Coade S.B. (1983). Purine transport and metabolism in man: The effect of exercise on concentrations of purine bases, nucleosides and nucleotides in plasma, urine, leucocytes and erythrocytes. Clin. Sci..

[36] Hellsten-Westing Y., Sollevi A., Sjodin B. (1991). Plasma accumulation of hypoxanthine, uric acid and creatine kinase following exhausting runs of differing durations in man. Eur. J. Appl. Physiol. Occup. Physiol..

[37] Ji L.L. (1996). Exercise, oxidative stress, and antioxidants. Am. J. Sports Med..

[38] Vincent H.K., Taylor A.G. (2006). Biomarkers and potential mechanisms of obesity-induced oxidant stress in humans. Int. J. Obes..

[39] Wolin M.S., Ahmad M., Gupte S.A. (2005). The sources of oxidative stress in the vessel wall. Kidney Int..

[40] Konior A., Schramm A., Czesnikiewicz-Guzik M., Guzik T.J. (2014). NADPH oxidases in vascular pathology. Antioxid. Redox Signal..

[41] Kaminski K.A., Bonda T.A., Korecki J., Musial W.J. (2002). Oxidative stress and neutrophil activation--the two keystones of ischemia/reperfusion injury. Int. J. Cardiol..

[42] Wheatcroft S.B., Williams I.L., Shah A.M., Kearney M.T. (2003). Pathophysiological implications of insulin resistance on vascular endothelial function. Diabet. Med..

[43] Cai H., Harrison D.G. (2000). Endothelial dysfunction in cardiovascular diseases: The role of oxidant stress. Circ. Res..

[44] Schiffrin E.L., Canadian Institutes of Health Research Multidisciplinary Research Group on Hypertension (2002). Beyond blood pressure: The endothelium and atherosclerosis progression. Am. J. Hypertens..

[45] Dandona P., Kumar V., Aljada A., Ghanim H., Syed T., Hofmayer D., Mohanty P., Tripathy D., Garg R. (2003). Angiotensin II receptor blocker valsartan suppresses reactive oxygen species generation in leukocytes, nuclear factor-kappa B, in mononuclear cells of normal subjects: Evidence of an antiinflammatory action. J. Clin. Endocrinol. Metab..

[46] Frisbee J.C., Maier K.G., Stepp D.W. (2002). Oxidant stress-induced increase in myogenic activation of skeletal muscle resistance arteries in obese Zucker rats. Am. J. Physiol. Heart Circ. Physiol..

[47] de Mello A.H., Costa A.B., Engel J.D.G., Rezin G.T. (2018). Mitochondrial dysfunction in obesity. Life Sci..

[48] Bogacka I., Xie H., Bray G.A., Smith S.R. (2005). Pioglitazone induces mitochondrial biogenesis in human subcutaneous adipose tissue in vivo. Diabetes.

[49] Martinez J.A. (2006). Mitochondrial oxidative stress and inflammation: An slalom to obesity and insulin resistance. J. Physiol. Biochem..

[50] Patel C., Ghanim H., Ravishankar S., Sia C.L., Viswanathan P., Mohanty P., Dandona P. (2007). Prolonged reactive oxygen species generation and nuclear factor-kappaB activation after a high-fat, high-carbohydrate meal in the obese. J. Clin. Endocrinol. Metab..

[51] Vincent H.K., Bourguignon C.M., Taylor A.G. (2010). Relationship of the dietary phytochemical index to weight gain, oxidative stress and inflammation in overweight young adults. J. Hum. Nutr. Diet..

[52] Kneepkens C.M., Lepage G., Roy C.C. (1994). The potential of the hydrocarbon breath test as a measure of lipid peroxidation. Free Radic. Biol. Med..

[53] Phillips M., Cataneo R.N., Greenberg J., Gunawardena R., Naidu A., Rahbari-Oskoui F. (2000). Effect of age on the breath methylated alkane contour, a display of apparent new markers of oxidative stress. J. Lab. Clin. Med..

[54] Cozzolino R., De Giulio B., Marena P., Martignetti A., Gunther K., Lauria F., Russo P., Stocchero M., Siani A. (2017). Urinary volatile organic compounds in overweight compared to normal-weight children: Results from the Italian I.Family cohort. Sci. Rep..

[55] Kistler M., Muntean A., Szymczak W., Rink N., Fuchs H., Gailus-Durner V., Wurst W., Hoeschen C., Klingenspor M., Hrabe de Angelis M. (2016). Diet-induced and mono-genetic obesity alter volatile organic compound signature in mice. J. Breath Res..

[56] Kistler M., Szymczak W., Fedrigo M., Fiamoncini J., Hollriegl V., Hoeschen C., Klingenspor M., Hrabe de Angelis M., Rozman J. (2014). Effects of diet-matrix on volatile organic compounds in breath in diet-induced obese mice. J. Breath Res..

[57] Alkhouri N., Eng K., Cikach F., Patel N., Yan C., Brindle A., Rome E., Hanouneh I., Grove D., Lopez R. (2015). Breathprints of childhood obesity: Changes in volatile organic compounds in obese children compared with lean controls. Pediatr. Obes..

[58] Alkhouri N., Cikach F., Eng K., Moses J., Patel N., Yan C., Hanouneh I., Grove D., Lopez R., Dweik R. (2014). Analysis of breath volatile organic compounds as a noninvasive tool to diagnose nonalcoholic fatty liver disease in children. Eur. J. Gastroenterol. Hepatol..

[59] Uchikawa M., Kato M., Nagata A., Sanada S., Yoshikawa Y., Tsunematsu Y., Sato M., Suzuki T., Hashidume T., Watanabe K. (2020). Elevated levels of proinflammatory volatile metabolites in feces of high fat diet fed KK-A(y) mice. Sci. Rep..

[60] Klemenz A.C., Meyer J., Ekat K., Bartels J., Traxler S., Schubert J.K., Kamp G., Miekisch W., Peters K. (2019). Differences in the Emission of Volatile Organic Compounds (VOCs) between Non-Differentiating and Adipogenically Differentiating Mesenchymal Stromal/Stem Cells from Human Adipose Tissue. Cells.

[61] Dragonieri S., Porcelli F., Longobardi F., Carratu P., Aliani M., Ventura V.A., Tutino M., Quaranta V.N., Resta O., de Gennaro G. (2015). An electronic nose in the discrimination of obese patients with and without obstructive sleep apnoea. J. Breath Res..

[62] Rowan D.D. (2011). Volatile metabolites. Metabolites.

[63] Longo V., Forleo A., Provenzano S.P., Coppola L., Zara V., Ferramosca A., Siciliano P., Capone S. (2018). HS-SPME-GC-MS metabolomics approach for sperm quality evaluation by semen volatile organic compounds (VOCs) analysis. Biomed. Phys. Eng. Express.

[64] Baranska A., Smolinska A., Boots A.W., Dallinga J.W., van Schooten F.J. (2015). Dynamic collection and analysis of volatile organic compounds from the headspace of cell cultures. J. Breath Res..

[65] (2014). Sohrabi. Mohsen; Zhang. Li; Zhang. Kai; Ahmetagic. Adnan; Wei. Ming, Q. Volatile Organic Compounds as Novel Markers for the Detection of Bacterial Clin. Microbial..

[66] Forleo A., Capone S., Longo V., Casino F., Radogna A.V., Siciliano P., Massaro M., Scoditti E., Calabriso N., Carluccio M.A. Evaluation of the Volatile Organic Compounds Released from Peripheral Blood Mononuclear Cells and THP1 Cells Under Normal and Proinflammatory Conditions. Proceedings of the AISEM Annual Conference on Sensors and Microsystems 2017.

[67] Khalid T., Aggio R., White P., De Lacy Costello B., Persad R., Al-Kateb H., Jones P., Probert C.S., Ratcliffe N. (2015). Urinary Volatile Organic Compounds for the Detection of Prostate Cancer. PLoS ONE.

[68] Thriumani R., Zakaria A., Hashim Y.Z.H., Jeffree A.I., Helmy K.M., Kamarudin L.M., Omar M.I., Shakaff A.Y.M., Adom A.H., Persaud K.C. (2018). A study on volatile organic compounds emitted by in-vitro lung cancer cultured cells using gas sensor array and SPME-GCMS. BMC Cancer.

[69] Ambrosone C.B. (2000). Oxidants and antioxidants in breast cancer. Antioxid. Redox Signal..

[70] Hakim M., Broza Y.Y., Barash O., Peled N., Phillips M., Amann A., Haick H. (2012). Volatile organic compounds of lung cancer and possible biochemical pathways. Chem. Rev..

[71] Watanabe M. (1998). Polymorphic CYP genes and disease predisposition--what have the studies shown so far?. Toxicol. Lett..

[72] Murray G.I. (2000). The role of cytochrome P450 in tumour development and progression and its potential in therapy. J. Pathol..

[73] Chen S. (1998). Aromatase and breast cancer. Front. Biosci..

[74] Terelius Y., Ingelman-Sundberg M. (1986). Metabolism of n-pentane by ethanol-inducible cytochrome P-450 in liver microsomes and reconstituted membranes. Eur. J. Biochem..

[75] Kohlmuller D., Kochen W. (1993). Is n-pentane really an index of lipid peroxidation in humans and animals? A methodological reevaluation. Anal. Biochem..

[76] Risby T.H., Sehnert S.S. (1999). Clinical application of breath biomarkers of oxidative stress status. Free Radic. Biol. Med..

[77] Marchitti S.A., Brocker C., Stagos D., Vasiliou V. (2008). Non-P450 aldehyde oxidizing enzymes: The aldehyde dehydrogenase superfamily. Expert Opin. Drug Metab. Toxicol..

[78] Rahman I., van Schadewijk A.A., Crowther A.J., Hiemstra P.S., Stolk J., MacNee W., De Boer W.I. (2002). 4-Hydroxy-2-nonenal, a specific lipid peroxidation product, is elevated in lungs of patients with chronic obstructive pulmonary disease. Am. J. Respir. Crit. Care Med..

[79] Vaz A.D., Coon M.J. (1987). Hydrocarbon formation in the reductive cleavage of hydroperoxides by cytochrome P-450. Proc. Natl. Acad. Sci. USA.

[80] Buszewski B., Kesy M., Ligor T., Amann A. (2007). Human exhaled air analytics: Biomarkers of diseases. Biomed. Chromatogr..

[81] Gordon S.M., Szidon J.P., Krotoszynski B.K., Gibbons R.D., O’Neill H.J. (1985). Volatile organic compounds in exhaled air from patients with lung cancer. Clin. Chem..

[82] Hakim M., Billan S., Tisch U., Peng G., Dvrokind I., Marom O., Abdah-Bortnyak R., Kuten A., Haick H. (2011). Diagnosis of head-and-neck cancer from exhaled breath. Br. J. Cancer.

[83] Horvath I., Lazar Z., Gyulai N., Kollai M., Losonczy G. (2009). Exhaled biomarkers in lung cancer. Eur. Respir. J..

[84] Branton P.J., McAdam K.G., Winter D.B., Liu C., Duke M.G., Proctor C.J. (2011). Reduction of aldehydes and hydrogen cyanide yields in mainstream cigarette smoke using an amine functionalised ion exchange resin. Chem. Cent. J..

[85] Ahotupa M., Bussacchini-Griot V., Bereziat J.C., Camus A.M., Bartsch H. (1987). Rapid oxidative stress induced by N-nitrosamines. Biochem. Biophys. Res. Commun..

[86] Kang J.O., Slater G., Aufses A.H., Cohen G. (1988). Production of ethane by rats treated with the colon carcinogen, 1,2-dimethylhydrazine. Biochem. Pharmacol..

[87] Burdock G., Fenaroli G. (2010). Fenaroli’s Handbook of Flavor Ingredients..

[88] Yannai S. (2004). Dictionary of Food Compounds with CD-ROM: Additives, Flavors, and Ingredients.

[89] Murray R., Granner D., Mayes P., Rodwell V. (2006). Harper’s Illustrated Biochemistry.

[90] Smith D., Wang T., Spanel P. (2002). On-line, simultaneous quantification of ethanol, some metabolites and water vapour in breath following the ingestion of alcohol. Physiol. Meas..

[91] Lagg A., Taucher J., Lindinger W. (1994). Application of proton transfer reactions to gas analysis. Int. J. Mass Spectrom. Ion Process..

[92] Xu Z.Q., Broza Y.Y., Ionsecu R., Tisch U., Ding L., Liu H., Song Q., Pan Y.Y., Xiong F.X., Gu K.S. (2013). A nanomaterial-based breath test for distinguishing gastric cancer from benign gastric conditions. Br. J. Cancer.

[93] Kumar S., Mohan A., Guleria R. (2006). Biomarkers in cancer screening, research and detection: Present and future: A review. Biomarkers.

[94] Riemenschneider W., Bolt H. (2005). Esters, Organic.

[95] Halliwell B., Gutteridge J.M., Cross C.E. (1992). Free radicals, antioxidants, and human disease: Where are we now?. J. Lab. Clin. Med..

[96] Guengerich F.P., Shimada T. (1991). Oxidation of toxic and carcinogenic chemicals by human cytochrome P-450 enzymes. Chem. Res. Toxicol..

[97] Greenberg M. (1999). Toxicological Review of Acetonitrile.

[98] Shibamoto T. (2006). Analytical methods for trace levels of reactive carbonyl compounds formed in lipid peroxidation systems. J. Pharm. Biomed. Anal..

[99] Sies H. (1997). Oxidative stress: Oxidants and antioxidants. Exp. Physiol..

[100] Peng G., Hakim M., Broza Y.Y., Billan S., Abdah-Bortnyak R., Kuten A., Tisch U., Haick H. (2010). Detection of lung, breast, colorectal, and prostate cancers from exhaled breath using a single array of nanosensors. Br. J. Cancer.

[101] Kasapovic J., Pejic S., Todorovic A., Stojiljkovic V., Pajovic S.B. (2008). Antioxidant status and lipid peroxidation in the blood of breast cancer patients of different ages. Cell Biochem. Funct..

[102] Mense S.M., Remotti F., Bhan A., Singh B., El-Tamer M., Hei T.K., Bhat H.K. (2008). Estrogen-induced breast cancer: Alterations in breast morphology and oxidative stress as a function of estrogen exposure. Toxicol. Appl. Pharmacol..

[103] Phillips M., Cataneo R.N., Ditkoff B.A., Fisher P., Greenberg J., Gunawardena R., Kwon C.S., Tietje O., Wong C. (2006). Prediction of breast cancer using volatile biomarkers in the breath. Breast Cancer Res. Treat..

[104] Ebeler S.E., Clifford A.J., Shibamoto T. (1997). Quantitative analysis by gas chromatography of volatile carbonyl compounds in expired air from mice and human. J. Chromatogr. B Biomed. Sci. Appl..

[105] Li J., Peng Y., Liu Y., Li W., Jin Y., Tang Z., Duan Y. (2014). Investigation of potential breath biomarkers for the early diagnosis of breast cancer using gas chromatography-mass spectrometry. Clin. Chim. Acta.

[106] Haick H., Broza Y.Y., Mochalski P., Ruzsanyi V., Amann A. (2014). Assessment, origin, and implementation of breath volatile cancer markers. Chem. Soc. Rev..

[107] Kneepkens C.M., Ferreira C., Lepage G., Roy C.C. (1992). The hydrocarbon breath test in the study of lipid peroxidation: Principles and practice. Clin. Investig. Med..

[108] Liu Y., Li W., Duan Y. (2019). Effect of H_2_O_2_ induced oxidative stress (OS) on volatile organic compounds (VOCs) and intracellular metabolism in MCF-7 breast cancer cells. J. Breath Res..

[109] Cikach F.S., Dweik R.A. (2012). Cardiovascular biomarkers in exhaled breath. Prog. Cardiovasc. Dis..

[110] Stevens J.F., Maier C.S. (2008). Acrolein: Sources, metabolism, and biomolecular interactions relevant to human health and disease. Mol. Nutr. Food Res..

[111] Moghe A., Ghare S., Lamoreau B., Mohammad M., Barve S., McClain C., Joshi-Barve S. (2015). Molecular mechanisms of acrolein toxicity: Relevance to human disease. Toxicol. Sci..

[112] Clemens M.R., Remmer H., Waller H.D. (1984). Phenylhydrazine-induced lipid peroxidation of red blood cells in vitro and in vivo: Monitoring by the production of volatile hydrocarbons. Biochem. Pharmacol..

[113] Kessler W., Remmer H. (1990). Generation of volatile hydrocarbons from amino acids and proteins by an iron/ascorbate/GSH system. Biochem. Pharmacol..

[114] Edson K.Z., Rettie A.E. (2013). CYP4 enzymes as potential drug targets: Focus on enzyme multiplicity, inducers and inhibitors, and therapeutic modulation of 20-hydroxyeicosatetraenoic acid (20-HETE) synthase and fatty acid omega-hydroxylase activities. Curr. Top. Med. Chem..

[115] Lieber C.S. (1997). Cytochrome P-4502E1: Its physiological and pathological role. Physiol. Rev..

[116] Raucy J.L., Lasker J.M., Kraner J.C., Salazar D.E., Lieber C.S., Corcoran G.B. (1991). Induction of cytochrome P450IIE1 in the obese overfed rat. Mol. Pharmacol..

[117] Weltman M.D., Farrell G.C., Hall P., Ingelman-Sundberg M., Liddle C. (1998). Hepatic cytochrome P450 2E1 is increased in patients with nonalcoholic steatohepatitis. Hepatology.

[118] Robertson G., Leclercq I., Farrell G.C. (2001). Nonalcoholic steatosis and steatohepatitis. II. Cytochrome P-450 enzymes and oxidative stress. Am. J. Physiol. Gastrointest. Liver Physiol..

[119] Aghdassi E., Allard J.P. (2000). Breath alkanes as a marker of oxidative stress in different clinical conditions. Free Radic. Biol. Med..

[120] Bernhard D., Wang X.L. (2007). Smoking, oxidative stress and cardiovascular diseases-do anti-oxidative therapies fail?. Curr. Med. Chem..

[121] Allard J.P., Royall D., Kurian R., Muggli R., Jeejeebhoy K.N. (1994). Effects of beta-carotene supplementation on lipid peroxidation in humans. Am. J. Clin. Nutr..

[122] Gelmont D., Stein R.A., Mead J.F. (1981). The bacterial origin of rat breath pentane. Biochem. Biophys. Res. Commun..

[123] Smith U., Hammersten J., Bjorntorp P., Kral J.G. (1979). Regional differences and effect of weight reduction on human fat cell metabolism. Eur. J. Clin. Investig..

[124] Mittelman S.D., Van Citters G.W., Kirkman E.L., Bergman R.N. (2002). Extreme insulin resistance of the central adipose depot in vivo. Diabetes.

[125] Meyer J., Salamon A., Mispagel S., Kamp G., Peters K. (2018). Energy metabolic capacities of human adipose-derived mesenchymal stromal cells in vitro and their adaptations in osteogenic and adipogenic differentiation. Exp. Cell Res..

[126] Drolet R., Richard C., Sniderman A.D., Mailloux J., Fortier M., Huot C., Rheaume C., Tchernof A. (2008). Hypertrophy and hyperplasia of abdominal adipose tissues in women. Int. J. Obes..

[127] Jo J., Gavrilova O., Pack S., Jou W., Mullen S., Sumner A.E., Cushman S.W., Periwal V. (2009). Hypertrophy and/or Hyperplasia: Dynamics of Adipose Tissue Growth. PLoS Comput. Biol..

[128] Spalding K.L., Arner E., Westermark P.O., Bernard S., Buchholz B.A., Bergmann O., Blomqvist L., Hoffstedt J., Naslund E., Britton T. (2008). Dynamics of fat cell turnover in humans. Nature.

[129] Zeve D., Tang W., Graff J. (2009). Fighting fat with fat: The expanding field of adipose stem cells. Cell Stem Cell.

[130] Tsushima Y., Nishizawa H., Tochino Y., Nakatsuji H., Sekimoto R., Nagao H., Shirakura T., Kato K., Imaizumi K., Takahashi H. (2013). Uric acid secretion from adipose tissue and its increase in obesity. J. Biol. Chem..

[131] Kushch I., Arendacka B., Stolc S., Mochalski P., Filipiak W., Schwarz K., Schwentner L., Schmid A., Dzien A., Lechleitner M. (2008). Breath isoprene--aspects of normal physiology related to age, gender and cholesterol profile as determined in a proton transfer reaction mass spectrometry study. Clin. Chem. Lab. Med..

[132] Simonen P., Kotronen A., Hallikainen M., Sevastianova K., Makkonen J., Hakkarainen A., Lundbom N., Miettinen T.A., Gylling H., Yki-Jarvinen H. (2011). Cholesterol synthesis is increased and absorption decreased in non-alcoholic fatty liver disease independent of obesity. J. Hepatol..

[133] Salerno-Kennedy R., Cashman K.D. (2005). Potential applications of breath isoprene as a biomarker in modern medicine: A concise overview. Wien. Klin. Wochenschr..

[134] Miekisch W., Schubert J.K., Noeldge-Schomburg G.F. (2004). Diagnostic potential of breath analysis--focus on volatile organic compounds. Clin. Chim. Acta.

[135] Franzese A., Vajro P., Argenziano A., Puzziello A., Iannucci M.P., Saviano M.C., Brunetti F., Rubino A. (1997). Liver involvement in obese children. Ultrasonography and liver enzyme levels at diagnosis and during follow-up in an Italian population. Dig. Dis. Sci..

[136] Schwimmer J.B., Deutsch R., Kahen T., Lavine J.E., Stanley C., Behling C. (2006). Prevalence of fatty liver in children and adolescents. Pediatrics.

[137] Hecht S.S. (2003). Tobacco carcinogens, their biomarkers and tobacco-induced cancer. Nat. Rev. Cancer.

[138] Ayala A., Munoz M.F., Arguelles S. (2014). Lipid peroxidation: Production, metabolism, and signaling mechanisms of malondialdehyde and 4-hydroxy-2-nonenal. Oxid. Med. Cell. Longev..

[139] Chien P.J., Suzuki T., Tsujii M., Ye M., Minami I., Toda K., Otsuka H., Toma K., Arakawa T., Araki K. (2017). Biochemical Gas Sensors (Biosniffers) Using Forward and Reverse Reactions of Secondary Alcohol Dehydrogenase for Breath Isopropanol and Acetone as Potential Volatile Biomarkers of Diabetes Mellitus. Anal. Chem..

[140] Capurso C., Capurso A. (2012). From excess adiposity to insulin resistance: The role of free fatty acids. Vascul. Pharmacol..

[141] Ouchi N., Parker J.L., Lugus J.J., Walsh K. (2011). Adipokines in inflammation and metabolic disease. Nat. Rev. Immunol..

[142] Grabowska-Polanowska B., Skowron M., Miarka P., Pietrzycka A., Sliwka I. (2017). The application of chromatographic breath analysis in the search of volatile biomarkers of chronic kidney disease and coexisting type 2 diabetes mellitus. J. Chromatogr. B Analyt. Technol. Biomed. Life Sci..

[143] Leiherer A., Geiger K., Muendlein A., Drexel H. (2014). Hypoxia induces a HIF-1alpha dependent signaling cascade to make a complex metabolic switch in SGBS-adipocytes. Mol. Cell. Endocrinol..

[144] Solga S.F., Alkhuraishe A., Cope K., Tabesh A., Clark J.M., Torbenson M., Schwartz P., Magnuson T., Diehl A.M., Risby T.H. (2006). Breath biomarkers and non-alcoholic fatty liver disease: Preliminary observations. Biomarkers.

[145] Raman M., Ahmed I., Gillevet P.M., Probert C.S., Ratcliffe N.M., Smith S., Greenwood R., Sikaroodi M., Lam V., Crotty P. (2013). Fecal microbiome and volatile organic compound metabolome in obese humans with nonalcoholic fatty liver disease. Clin. Gastroenterol. Hepatol..

